# A Multidisciplinary Approach to mHealth: Developing a Protocol of Prehabilitation App for Abdominal Wall Hernia Surgery

**DOI:** 10.3390/mps9050139

**Published:** 2026-09-23

**Authors:** José Luis Gil Delgado, Carlos Rangel Cascajosa, Alejandro Sánchez Arteaga, Luis Tallón Aguilar, Borja Sañudo Corrales

**Affiliations:** 1 Department of Physical Education and Sports, University of Seville, 41900 Seville, Spain; crangel@us.es (C.R.C.); bsancour@us.es (B.S.C.); 2 Department of General Surgery, Hospital Universitario Virgen del Rocío, 41013 Seville, Spain; alejandros.arteaga@gmail.com (A.S.A.); ltallon@us.es (L.T.A.)

**Keywords:** prehabilitation, abdominal wall hernia, mobile health (mHealth), personalised exercise programme

## Abstract

Introduction: Abdominal wall hernias (AWH) represent distinct pathological structural defects, with incisional hernias occurring as late surgical failures following laparotomy, and prehabilitation has shown promise in improving functional recovery. However, access to hospital-supervised programmes is frequently limited. Methodology: A multidisciplinary team developed the HERNIACare Lab mobile application, integrating clinical surgery, biomedical engineering, and sports science expertise. The development process involved clinical evidence synthesis, structured exercise protocol design (FITT-VP principles), software architecture development, and system integration. Results: The application incorporates four primary functional modules: baseline isometric strength stratification, programme scheduling, progressive multicomponent training, and post-session exertion and symptom monitoring. It features sex-specific tertile-based stratification, progressive overload principles with intra-abdominal safety constraints, and an accessible interface. Discussion: HERNIACare Lab addresses practical barriers to prehabilitation by delivering a structured home-based intervention tailored to baseline functional status. Conclusions: The application provides a clinically informed, accessible framework for remote prehabilitation, with clinical safety, usability, and effectiveness to be evaluated in prospective clinical trials.

## 1. What Is Known

Structured exercise can enhance functional capacity and support postoperative recovery in surgical populations. Geographic, financial, and mobility barriers limit widespread participation in hospital-based, supervised prehabilitation programmes. Mobile health (mHealth) tools offer a scalable channel for remote guidance, yet existing surgical applications rarely incorporate baseline functional stratification or pathology-specific abdominal wall safety mechanisms.

## 2. What Is New

Introduces the design and architecture of HERNIACare Lab, a dedicated mHealth platform for abdominal wall hernia prehabilitation. Establishes a sex-specific tertile stratification model derived from reference isometric dynamometry to assign tailored entry-level training volumes and intensities. Integrates specific abdominal safety criteria, avoiding sustained high intra-abdominal pressure (e.g., Valsalva manoeuvres) while ensuring structured progressive overload and symptom-triggered monitoring. Outlines the technical, regulatory, and data governance frameworks required for clinical implementation under European medical device and data privacy legislation. Provides a fully reproducible exercise protocol detailing exercise typology, progression stages, rest intervals, and clinical stop criteria, alongside the prospective randomized controlled trial framework for clinical validation.

## 3. Introduction

Abdominal Wall Hernias (AWH) are distinct anatomical conditions characterized by the protrusion of intra-abdominal contents through an acquired or congenital defect in the musculoaponeurotic layer [1,2,3]. Primary ventral hernias arise spontaneously at sites of anatomical weakness, whereas incisional hernias represent late failures of fascial healing following prior abdominal surgery, frequently resulting in pain, mechanical impairment, and recurrent hospitalisations [1,2,3].

Unlike general prehabilitation populations, such as elective orthopaedic or general oncological cohorts, patients with abdominal wall defects present unique biomechanical challenges. In these patients, any increase in intra-abdominal pressure directly stresses the fascial boundaries of the defect. Consequently, physical preparation cannot rely on conventional resistance or high-intensity conditioning protocols. Prehabilitation must safely enhance core neuromuscular recruitment and physical conditioning while avoiding excessive tensile strain or sustained pressure spikes across the abdominal defect.

Optimising the preoperative condition of patients through structured physical preparation, commonly referred to as prehabilitation, has gained relevance as a strategy to improve surgical outcomes, reduce complications, and facilitate recovery [4,5]. Despite its demonstrated benefits, access to supervised prehabilitation programmes remains limited, particularly for patients with mobility challenges or those residing far from specialised healthcare centres [6]. In this context, mobile health (mHealth) technologies represent a promising alternative, enabling remote, personalised, and continuous guidance tailored to individual needs [7,8].

While various health applications have been developed to support rehabilitation processes, most are generic or focused on other conditions [9,10]. To date, there is a notable lack of digital tools specifically designed to support preoperative training and postoperative monitoring in patients with AWH, a population that requires careful attention to abdominal integrity and exercise intensity.

This article describes the conceptualization and development of a mobile application (HERNIACare Lab) specifically designed to address this clinical gap. Developed in collaboration with biomedical engineers and abdominal wall surgeons from the Hospital Universitario Virgen del Rocío (Seville, Spain), the application integrates clinical expertise and technological innovation to deliver a user-centred, functional solution. The objective of this work is to outline the conceptual underpinnings, reproducible exercise mechanics, safety controls, data security infrastructure, and prospective clinical trial framework of the application, laying the groundwork for its future clinical validation.

## 4. Methodology

### 4.1. Multidisciplinary Team

The development of the HERNIACare Lab application was carried out by a multidisciplinary team bringing together expertise from biomedical engineering, clinical surgery, and sports science. Biomedical engineers from the University of Seville led the technical development, ensuring the digital infrastructure met usability, security, and adaptability standards. Abdominal wall surgeons from the specialised surgical unit at Hospital Universitario Virgen del Rocío (Seville, Spain) provided clinical oversight, defining patient needs, safety criteria, contraindicated movement patterns, and exercise suitability. In parallel, sport scientists from the Department of Physical Education and Sport at the University of Seville contributed to the design of evidence-based physical training protocols tailored to preoperative requirements. This collaborative framework enabled the integration of medical accuracy, digital functionality, and exercise science within a single platform.

### 4.2. Project Development Phases

The development of the application followed a structured, evidence-based approach articulated in four key phases:

Phase 1: Evidence synthesis and clinical rationale

Scientific literature addressing exercise interventions in abdominal surgery, trunk biomechanics, and exercise-induced intra-abdominal pressure was evaluated. While postoperative studies have investigated trunk stability and rehabilitation feasibility [11,12,13], prospective evidence in preoperative AWH remains sparse. Feasibility research, such as home-based trials in abdominal stoma and bulge cohorts, has documented challenges including suboptimal compliance (e.g., retention below 50%) [12]. These observations reinforced the necessity of designing an intuitive, low-barrier, and progressively monitored home intervention.

Phase 2: Training protocol design and abdominal integrity criteria

Based on the evidence gathered, specific training protocols were developed in collaboration with experts in sports science and clinical rehabilitation. These protocols were tailored to the needs of patients preparing for abdominal wall surgery, ensuring safety, feasibility, and progressive intensity. To protect the mechanical integrity of the abdominal wall defect, exercises were selected to avoid sustained Valsalva manoeuvres and high intra-abdominal peak pressure. Training prioritised dynamic, low-impact trunk muscle co-activation, closed-kinetic-chain stability, and low-load isometric holds. The training programme followed a linear periodisation model, whereby both the volume and intensity of the exercises were gradually increased over the intervention period [14]. The progressive increase in volume was primarily achieved by systematically augmenting the number of sets and repetitions prescribed each week. In parallel, exercise intensity was progressively advanced through the incorporation of more complex movement patterns or longer lever arms. To regulate and individualise this progression, a perceived exertion scale ranging from 0 (no effort) to 10 (maximal effort) was employed [15]. Whenever participants reported an exertion level of 4–5 out of 10 for a given exercise, the intensity was adjusted by modifying the technical complexity or external resistance, thereby promoting continuous adaptation and minimising the risk of performance plateaus. Conversely, reporting localized pain or acute discomfort prompted immediate reduction in loading.

Phase 3: Application architecture and functional design

The third phase focused on the digital translation of the clinical protocols into an interactive and personalised mobile application. The app was conceptualised not merely as a static programme delivery tool but as a dynamic platform capable of adapting to each patient’s initial physical condition. To this end, several core components were developed: an initial assessment module to determine baseline functional status (Figure 1); a calendar and follow-up interface to promote continuity and adherence (Figure 2); the core training section, containing structured video-guided sessions (Figure 3); and a post-session self-assessment interface for users to report perceived exertion and difficulty, enabling iterative adaptation of the programme (Figure 4).

Phase 4: Software integration and alpha usability testing

The final phase, currently underway, involves the integration of the training protocols into the app’s operational framework. This includes synchronising exercise content, feedback mechanisms, and user interfaces to ensure functional coherence. Internal testing is being conducted to evaluate crash rates, synchronization stability, and interface accessibility among non-expert users, laying the groundwork for subsequent clinical evaluation through a prospective randomised controlled trial.

### 4.3. Detailed Exercise Protocol and Reproducibility Specifications

The HERNIACare Lab intervention consists of an 8-week home-based programme with 2 sessions per week on non-consecutive days, lasting 45 to 60 min per session:

Structure of each session:

1. Warm-up (8–10 min): Diaphragmatic breathing mechanics in supine and seated positions, low-velocity mobility (pelvic tilts, thoracic rotations, ankle and hip mobilization), and low-intensity aerobic activation (marching in place).
2. Main Conditioning Phase (30–40 min): 5 to 6 core exercises comprising:
  - 2 trunk neuromuscular stabilization exercises: wall-supported planks, bird-dog progressions, bridges, and modified dead bugs avoiding abdominal doming.
  - 2 functional resistance exercises: sit-to-stand/box squats, wall push-ups, elastic band rows, and step-ups.
  - 1 aerobic-metabolic interval: low-impact stepped intervals, seated cycling simulation, or continuous brisk marching.
3. Cool-down (5–7 min): Low-intensity walking, passive stretching of hip flexors and pectorals, and relaxation breathing.

Periodisation and Progression:

- Weeks 1–2 (Familiarisation & Activation): 2 sets of 8–10 repetitions (or 10–15 s isometric holds), 60–90 s rest between sets. Intensity target: RPE 3–4/10.
- Weeks 3–5 (Volume Progression): 3 sets of 10–12 repetitions (or 15–20 s holds), 60 s rest. Intensity target: RPE 4–6/10.
- Weeks 6–8 (Intensity & Biomechanical Progression): 3 sets of 12–15 repetitions (or 20–30 s holds) with increased lever arms or added elastic band resistance. Intensity target: RPE 6–7/10.

Safety Rules and Stop Criteria:

- Strict contraindication of the Valsalva manoeuvre: continuous, audible exhalation is cued during concentric/exertion phases.
- Exercise must be immediately interrupted if the patient experiences localized hernia pain > 3/10 on the VAS, sensation of acute fascial tearing, sudden hernia enlargement/irreducibility, dizziness, or shortness of breath.

### 4.4. Clinical and Technical Criteria for Application Design

The design of the application was guided by clinical and technical criteria aimed at ensuring the safety, effectiveness, and accessibility of the preoperative training programme. Clinically, the content was structured based on scientific evidence gathered during the initial phase of the project. These criteria informed the selection of exercises, their progressive intensity, and the definition of functional limits adapted to the physical conditions of surgical candidates. To ensure clinical appropriateness, specific criteria were defined regarding the duration, frequency, and type of exercises, with a focus on core activation, trunk muscle strengthening, and overall functional capacity enhancement. Consideration was also given to mobility restrictions and common comorbidities, which led to the development of low-impact, video-guided sessions with clear instructions, designed to promote adherence without compromising abdominal integrity.

From a technical perspective, the application was initially developed for the Android platform, due to its wider availability among older adults and individuals from diverse socioeconomic backgrounds. User-centred design principles were applied, with particular attention to usability for elderly users and those with limited digital literacy. The interface features clear visual elements, intuitive navigation, and immediate feedback, minimising technological barriers and facilitating autonomous use. Additionally, a modular and scalable architecture was prioritised, enabling future clinical and functional updates without disrupting the user experience.

### 4.5. Digital Tools, Technological Platforms, and Data Governance

The application was developed using Flutter (version 3.47; https://flutter.dev (accessed on 16 September 2026)), an open-source UI software development toolkit created by Google. Flutter enables cross-platform development from a single codebase using the Dart programming language. This choice enables a unified codebase to deliver a consistent user experience across Android and iOS devices, as well as web-based platforms. Flutter’s architecture facilitates efficient deployment and maintenance while ensuring visual and functional coherence across systems. The client application utilizes encrypted local storage strictly for transient session caching, interface configuration state, and offline video asset buffering to ensure uninterrupted performance during network drops. All persistent clinical and personal user records are transmitted and housed in an external, dedicated PostgreSQL relational database hosted within the European Union (Frankfurt, Germany) on ISO/IEC 27001-certified infrastructure. From a data protection standpoint, data handling complies with Regulation (EU) 2016/679 (General Data Protection Regulation, GDPR) and Spanish Organic Law 3/2018 (LOPDGDD). The University of Seville acts as the Data Controller. Processing of special category health data is legally grounded in Article 9(2)(j) of the GDPR (scientific research purposes) alongside explicit participant consent (Article 6(1)(a)). The system architecture incorporates the following safeguards:

- Pseudonymisation: All patient metrics and exercise logs are dissociated from direct nominative identifiers via randomly generated alphanumeric universal unique identifiers (UUIDs). The key linking UUIDs to patient identities is held exclusively by the clinical study coordinator on an isolated, firewalled hospital server.
- Encryption: Data in transit are encrypted via Transport Layer Security (TLS 1.3/HTTPS). Data at rest within the external database and local app sandbox are encrypted using Advanced Encryption Standard with 256-bit keys (AES-256).
- National Security Scheme (ENS): The platform configuration adheres to the Basic category requirements of the Spanish Esquema Nacional de Seguridad (ENS; Real Decreto 311/2022). A formal Data Protection Impact Assessment (DPIA) was completed in accordance with AEPD guidelines.
- Retention: Research data will be retained for five years post-study closure in conformity with biomedical research standards, after which records will be permanently expunged.

Medical Device Software (MDSW) Considerations:

Under Regulation (EU) 2017/745 (MDR), software providing individualised diagnostic stratification and therapeutic exercise intensity prescription for a pathological state qualifies as Medical Device Software (MDSW). Under MDR Annex VIII, Rule 11, software intended to provide information used to take decisions with therapeutic purposes is classified as Class IIa. The current software build is categorized as an Investigational Medical Device Software exclusively deployed within the regulatory boundaries of an authorized clinical research protocol (MDR Article 62), prior to formal CE mark conformity assessment.

## 5. Application Design and Functional Description

Module 1: Baseline Abdominal Wall Strength Stratification

The foundation of the application’s personalisation capability lies in the first module, which collects and integrates baseline strength data of the abdominal wall musculature. These data are obtained through standardised measurements conducted in outpatient surgical consultations using validated handheld dynamometric procedures (ActivForce 2) [16]. The stratification thresholds were derived from a reference cohort of 62 surgical candidates evaluated at the specialized Abdominal Wall Unit of Hospital Universitario Virgen del Rocío [16]. The cohort comprised 39 women and 23 men (mean age: 57.6 ± 11.2 years; mean body mass index: 31.4 ± 5.1 kg/m^2^) presenting with midline incisional hernias (EHS classification: midline M2–M4, width W2 [4–10 cm]). Patients with active systemic infections, acute hernia incarceration, or severe neurological disorders compromising voluntary muscle contraction were excluded. To define the training categories, the strength values collected were stratified by sex to account for physiological differences in muscle force production between men and women. Subsequently, statistical tertile distributions were calculated within each sex-specific dataset to establish clinically relevant thresholds. For women, the low training level corresponds to strength values below the 33rd percentile (<99.4 N), the medium level includes values between the 33rd and 66th percentiles (99.4–174.0 N), and the high level comprises values above the 66th percentile (>174.0 N). For men, the low level was defined as <155.8 N, the medium level as 155.8–245.9 N, and the high level as >245.9 N, following an analogous percentile-based approach. These thresholds represent empirical statistical tertile divisions calculated directly from the reference clinical database rather than algorithmic machine learning models. This division establishes an initial, physiologically proportionate training volume and exercise difficulty level. Given that these reference values were derived from a single-centre incisional hernia cohort, they serve as preliminary programmatic benchmarks that will require external calibration across wider clinical presentations (e.g., umbilical, inguinal, primary ventral hernias) during subsequent multi-centre trials. Overall, this approach ensures that each patient is assigned to an initial training programme tailored to their functional capacity, thereby reducing the risk of overload, facilitating adherence, and enhancing the individualisation of the intervention.

Module 2: Calendar and programme scheduling

The second module facilitates temporal organisation and adherence through an intuitive calendar interface. Patients are empowered to determine their programme start date and view their training schedule over the eight-week intervention period. The calendar dynamically displays assigned sessions, allowing patients to anticipate and prepare for each activity. Reminders and visual progress indicators are incorporated to reinforce engagement and provide a sense of achievement. The design of this module reflects principles of behavioural science, recognising that adherence in home-based programmes is closely linked to structure, predictability, and self-monitoring. By offering a user-friendly scheduling interface, the app supports habit formation and facilitates integration into daily routines.

Module 3: Individualised multicomponent training programme

The core of the application resides in its training module, where patients access their personalised exercise sessions. The app automatically loads the specific exercise progression matched to the user’s baseline strength tertile and current training week. Programmes span eight weeks and include two weekly sessions, each lasting approximately 45 to 60 min. The training approach is multicomponent, combining exercises targeting muscular strength, flexibility, mobility, and cardiovascular conditioning. The principle of progressive overload is applied throughout the eight-week period, with difficulty levels and volume gradually increasing in line with expected physiological adaptation [17]. Sessions are guided via high-quality video demonstrations, accompanied by verbal and visual instructions to ensure correct execution. All exercises incorporate explicit abdominal wall protection instructions: avoiding breath-holding, maintaining relaxed diaphragmatic respiration, and executing movements within comfortable pain-free margins.

Module 4: Session Feedback and Perceived Exertion Monitoring

To capture patient experience and support iterative refinement of training protocols, the application incorporates a post-session feedback module. Upon completing each session, users are prompted to report their perceived exertion on a standardised 0–10 scale, where 0 indicates no effort and 10 represents maximal effort [18]. In addition to exertion, patients are asked to rate the intensity of pain experienced in the herniated area using a visual analogue scale (VAS) ranging from 0 (no pain) to 10 (maximum pain) [19]. If a participant logs localized pain exceeding 3/10, or an RPE inconsistent with the assigned phase, the interface provides an automated safety recommendation to pause training and triggers an alert for clinical research team review prior to the next scheduled bout. In addition to the numerical rating, patients may submit free-text comments describing their experience, challenges, or suggestions. Overall, this fourth module closes the intervention loop by linking execution with reflection, enabling continuous learning and adjustment at both the individual and system levels.

## 6. Discussion

This study presents the design of a mobile application specifically developed to deliver a structured prehabilitation programme for patients undergoing abdominal wall hernia surgery. While digital health tools have gained traction in surgical contexts, few have addressed the unique needs of this patient population, in which abdominal integrity and trunk muscle conditioning are essential [20]. The present application introduces several innovations that distinguish it from existing digital prehabilitation tools, including a personalised training algorithm based on abdominal wall strength, a multicomponent training approach, and a user-centred interface tailored for accessibility.

Compared to previous interventions, this application aligns with recent efforts to bring prehabilitation into home-based digital formats [21]. For example, the “Be Prepared” app, evaluated in a multi-centre randomised controlled trial undergoing major elective surgery, demonstrated improved patient satisfaction. However, it did not yield clinically meaningful gains in physical function, potentially due to inadequate training intensity or the absence of personalised exercise programming [22]. In contrast, the current application includes objective muscle strength assessment to guide training assignment and applies progressive overload principles, two key strategies known to enhance training outcomes.

Similarly, although the PROTEGO MAXIMA app trial demonstrated the feasibility and safety of remote aerobic prehabilitation using wearable telemetry, it lacked a multicomponent approach and was primarily focused on improving cardiovascular fitness [23]. The present tool integrates flexibility, strength, mobility, and endurance exercises, thereby adhering more closely to evidence-based recommendations for surgical candidates.

Other digital solutions have attempted to incorporate multiple components or deliver multidisciplinary guidance. For example, Li et al. [24] developed an mHealth app including multicomponent training and lifestyle interventions. While the app demonstrated high usability, it was not specifically targeted at abdominal health and lacked stratification based on user characteristics. In contrast, our application is, to our knowledge, the first to tailor exercise prescriptions using actual trunk muscle strength data obtained from a clinical reference cohort. This approach enables a personalized experience based on functional capacity and sex-specific thresholds, thereby enhancing both relevance and efficacy.

Studies such as the HALT trial in parastomal bulging observed substantial adherence attrition (44% retention), underlining the necessity for structured, accessible, and user-friendly digital tools that balance progressive overload with biomechanical safety [12]. HERNIACare Lab incorporates sex-stratified entry levels, linear volume progression, and real-time symptom tracking to address these identified challenges.

Planned Clinical Validation Trial: To assess the clinical efficacy and feasibility of HERNIACare Lab, a prospective, two-arm, parallel-group randomised controlled trial (RCT) is planned at Hospital Universitario Virgen del Rocío:

- Design & Arms: Patients scheduled for elective abdominal wall hernia repair will be randomized 1:1 to either the HERNIACare Lab prehabilitation group (8 weeks of app-guided training) or a control group receiving standard preoperative advice.
- Primary Endpoint: Change in maximal isometric force (FIM) and rate of force development (RFD) during isometric sagittal and oblique trunk flexion from baseline to preoperative assessment (week 8), evaluated via standardized dynamometric assessment.
- Secondary Endpoints: Functional physical performance assessed via the 30-Second Chair Stand Test (30 s CST) and 6-Minute Walk Test (6MWT), hernia-related quality of life (HerQLes questionnaire), 30-day postoperative surgical complications (graded by Clavien–Dindo classification), hospital length of stay, and app adherence and usability metrics (SUS questionnaire and completion rates).
- Sample Size: Based on detecting clinically relevant differences in trunk neuromuscular parameters (alpha = 0.05, power = 80%), and accounting for an anticipated dropout rate, a total sample size of 66 patients (33 per group) will be recruited.
- Trial Registration: The protocol has been prepared for registration on ClinicalTrials.gov upon final institutional review board release.

Limitations and Future Directions: Several methodological considerations warrant acknowledgment. First, the baseline strength thresholds were derived from a single-centre convenience cohort restricted to incisional hernia patients [16]. While this provided an empirical basis for tertile allocation, external validity across diverse hernia phenotypes (e.g., small primary defects, massive complex defects) remains unconfirmed and will be addressed in future multi-centre studies. Second, the home-based setting depends entirely on autonomous patient adherence and subjective reporting without real-time objective biomechanical monitoring of exercise execution (e.g., via wearable inertial sensors or computer vision). Third, the current version lacks direct, synchronous clinician-in-the-loop oversight dashboards, functioning primarily via client-side alerts and asynchronous research notifications. Fourth, behavioural engagement strategies, such as gamification or peer support, were not implemented in the current version and may be considered in future iterations, given recent findings showing that social comparison-based gamification features can enhance physical activity, reduce sedentary behaviour, and improve psychosocial outcomes in mHealth users [25]. Finally, deployment beyond investigational research will require complete conformity assessment and technical documentation in compliance with MDR (EU) 2017/745 Class IIa requirements.

## 7. Conclusions

HERNIACare Lab offers an accessible, structured, and clinically guided digital prehabilitation framework specifically developed for abdominal wall hernia patients. By integrating sex-specific baseline strength stratification, progressive multicomponent exercises with intra-abdominal safety constraints, and post-session symptom monitoring, the platform seeks to overcome traditional accessibility barriers. Forthcoming randomized controlled trials will assess the application’s clinical efficacy, usability, and adherence profile in surgical candidates.

## Figures and Tables

**Figure 1:**
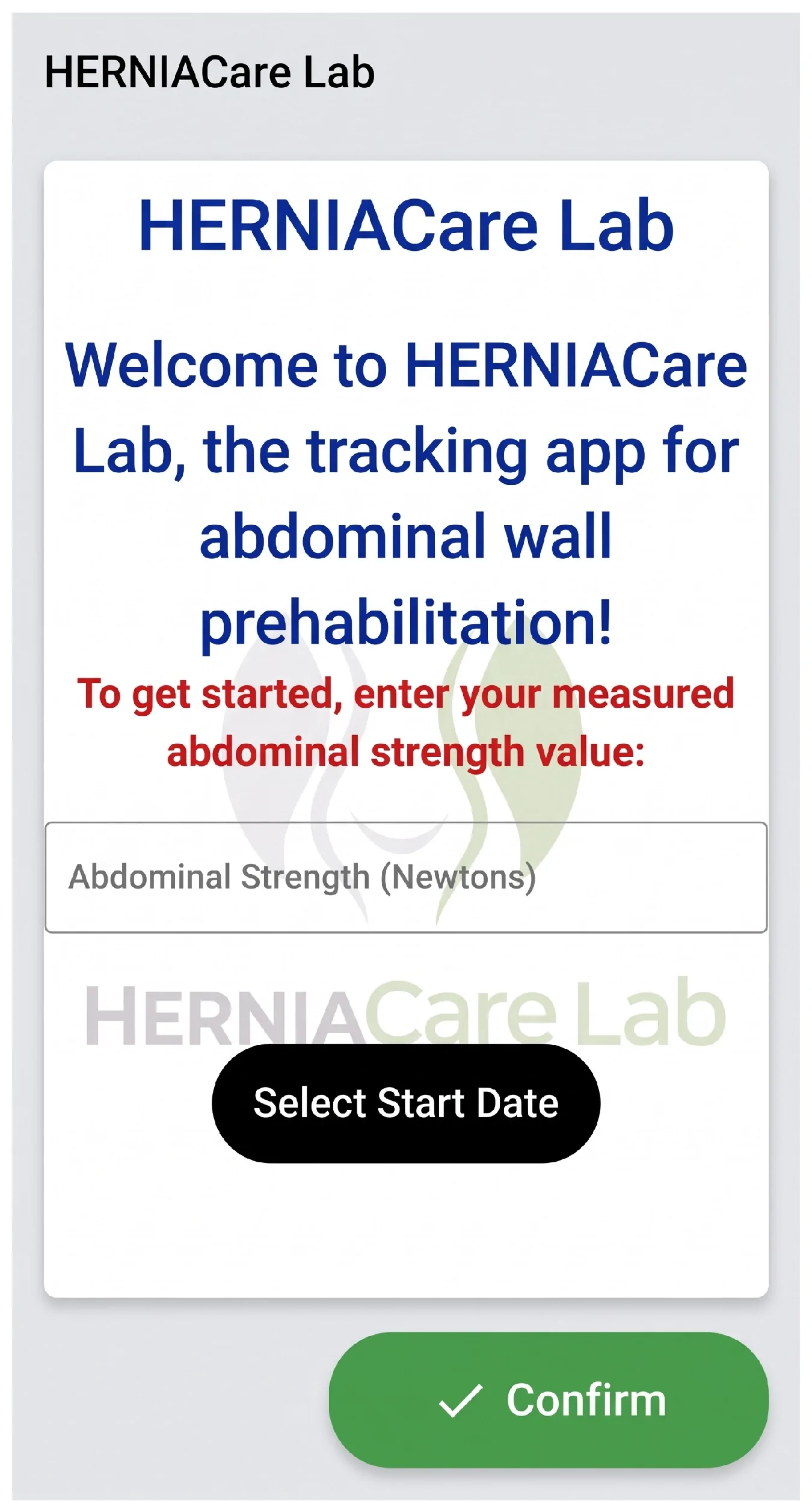
Initial assessment.

**Figure 2:**
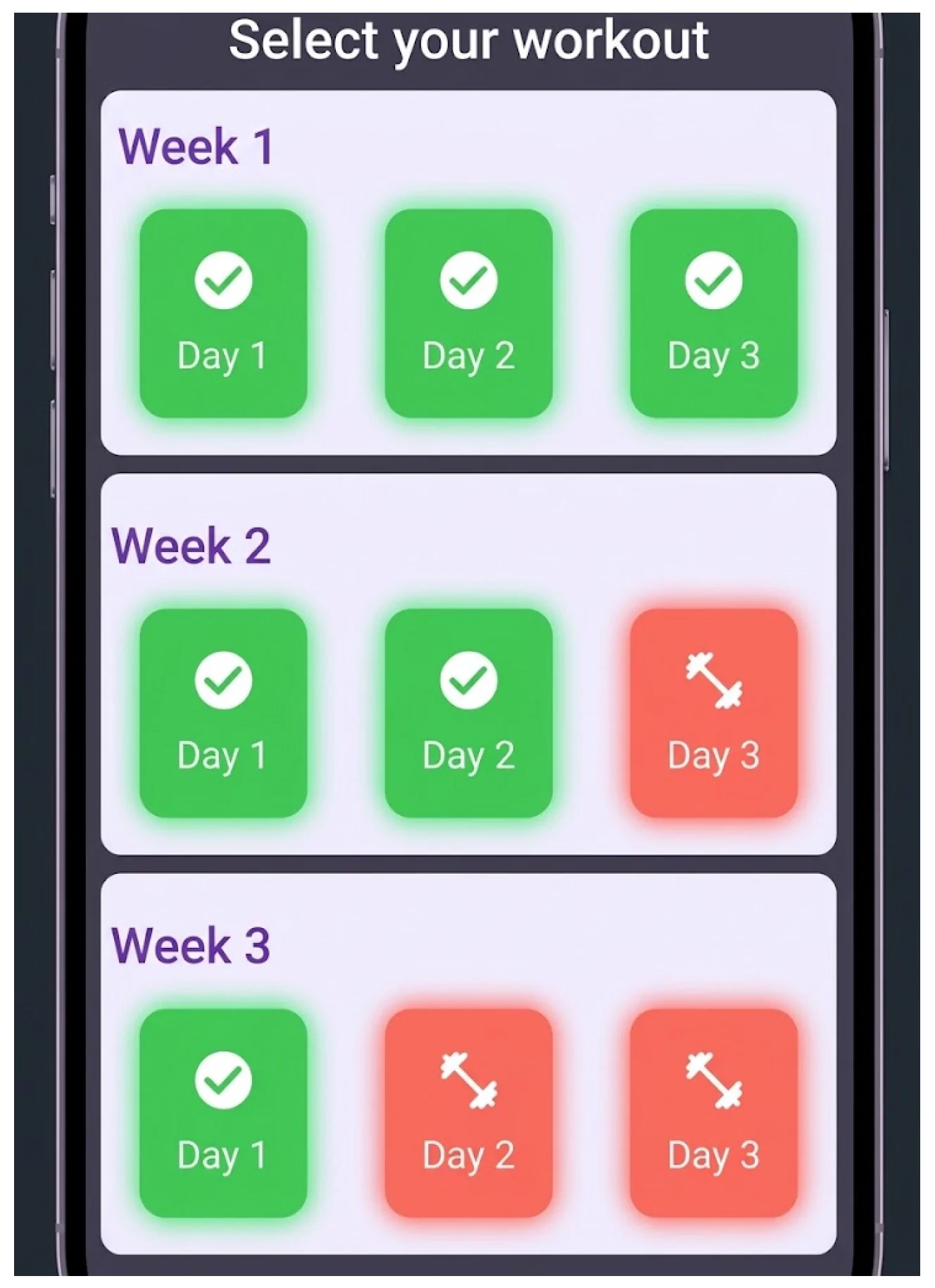
Calendar and follow-up.

**Figure 3:**
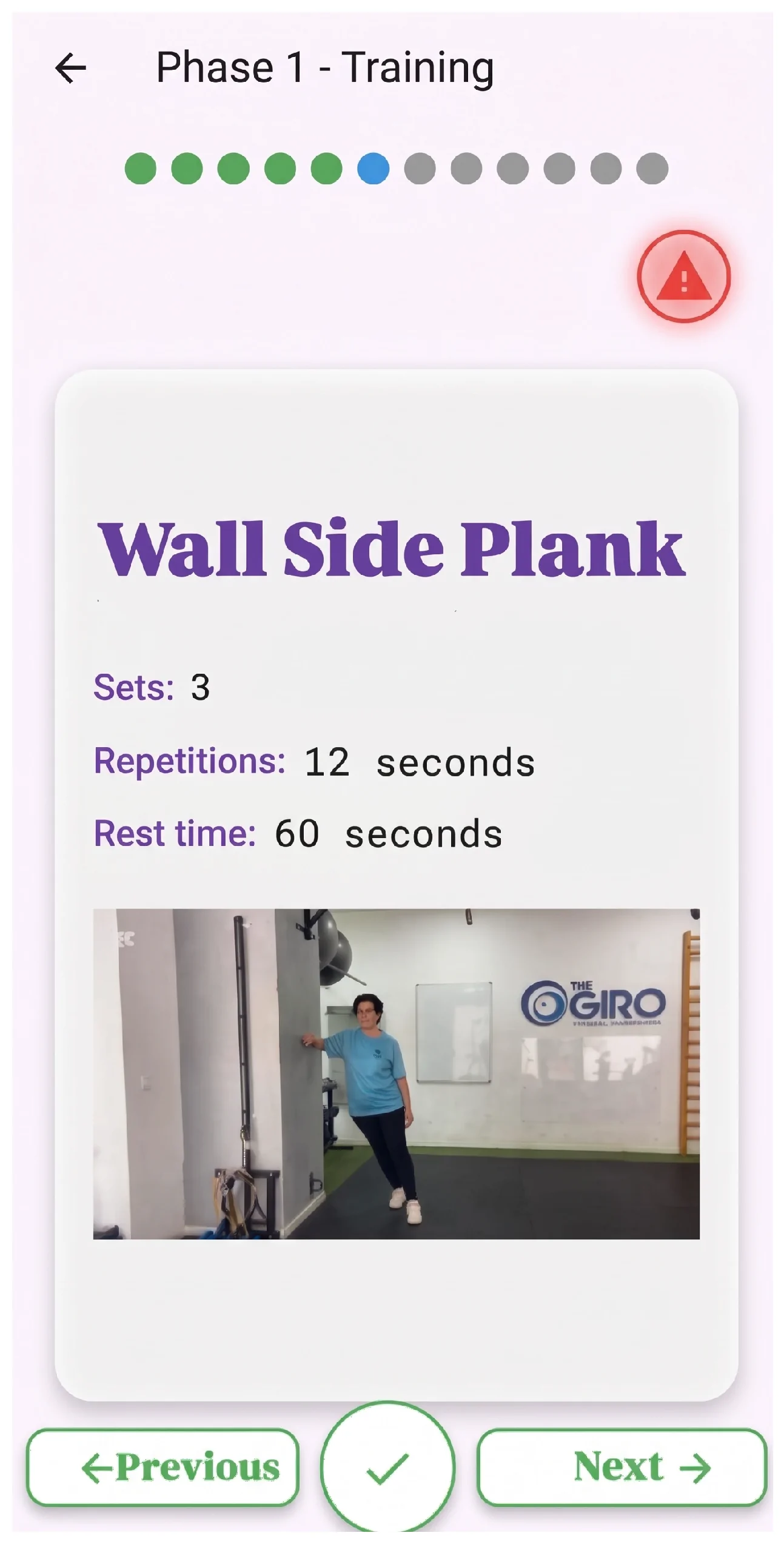
Training section. The progress bar at the top shows the exercises of the session: green dots indicate completed exercises, the blue dot the current exercise, and grey dots pending exercises. The red warning symbol allows the user to report symptoms or discomfort during the session. Each exercise card displays the prescribed sets, repetitions (or duration), and rest time, together with an instructional video.

**Figure 4:**
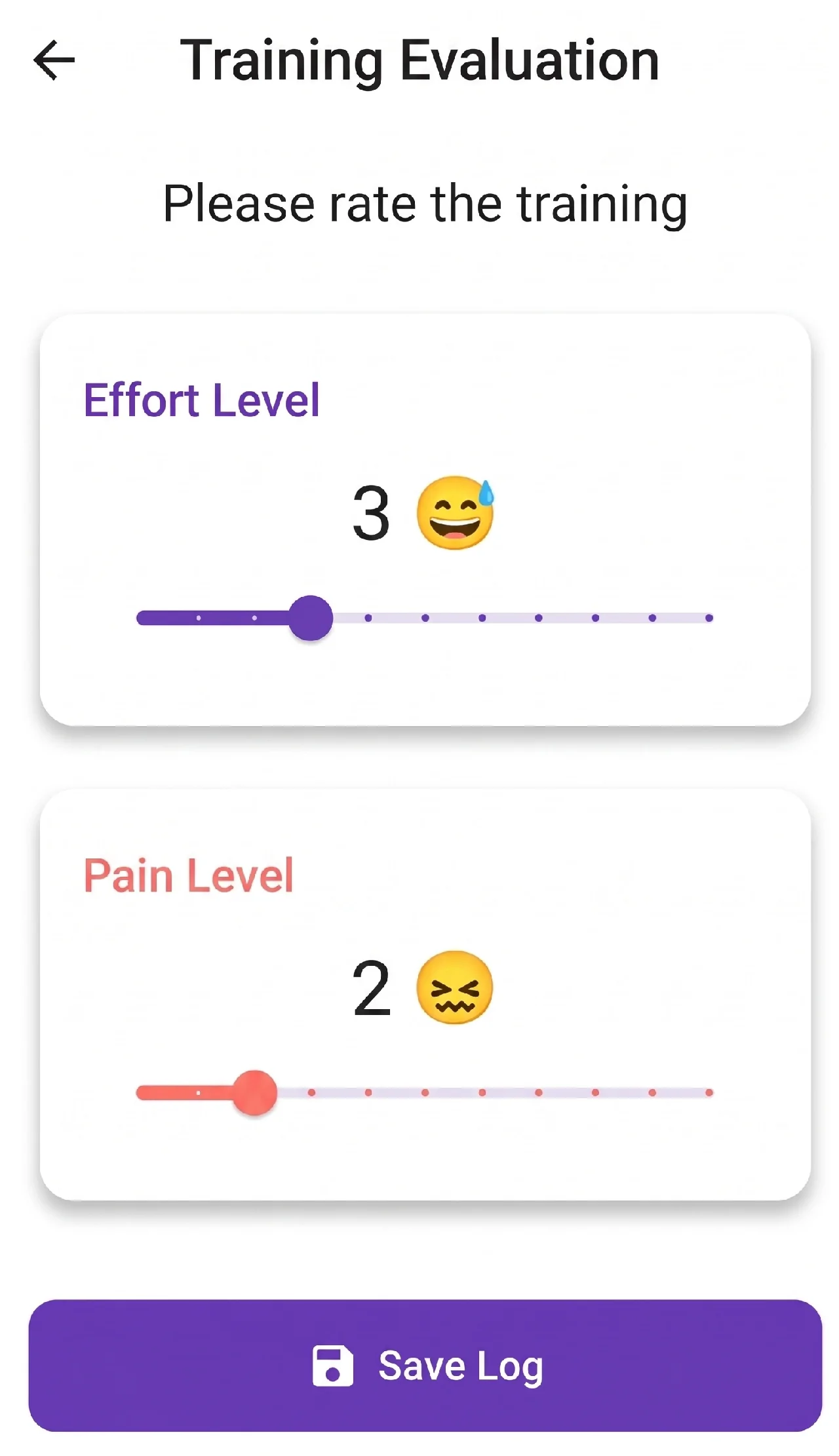
Post-session self-assessment. Users rate their perceived effort (0 = no effort to 10 = maximal effort) and pain in the herniated area (0 = no pain to 10 = maximum pain) using sliders; the emoji next to each value changes according to the selected score to provide intuitive visual feedback. The arrow at the top left returns to the previous screen, and the “Save Log” button stores the evaluation.

## Data Availability

The technical data and algorithmic architecture supporting the conclusions of this article are available from the corresponding author upon reasonable request. Clinical baseline reference datasets are restricted to preserve patient confidentiality.
